# Tuberculome à localisation inhabituelle: noyau caudé

**DOI:** 10.11604/pamj.2016.25.25.10446

**Published:** 2016-09-27

**Authors:** Maha Ait Berri, Abdelhadi Rouimi

**Affiliations:** 1 Service de Neurologie, Hôpital Militaire Moulay Ismail, Meknès, Maroc

**Keywords:** Tuberculome, Noyau caudé, Imagerie par résonance magnétique, Tuberculoma, caudate nucleus, magnetic resonance imaging MRI

## Image en médecine

Nous rapportons l'observation d'un patient de 30 ans qui a présenté un syndrome méningé fébrile. L'examen du LCR retrouvait une méningite lymphocytaire à 400 éléments blancs avec hypoglucorachie évoquant une méningite tuberculeuse. Une imagerie cérébrale (TDM puis IRM) (A, B) a été réalisée. Elle révéla la présence d'un tuberculome cérébral localisé au niveau du noyau caudé. Le patient a été mis sous traitement anti-bacillaire avec bonne évolution. Les tuberculomes constituent une complication rare des atteintes tuberculeuses du SNC. Leur siège de prédilection est la jonction substance blanche-grise. Leur diagnostic repose sur l'imagerie. Ils peuvent être uniques ou multiples. Au scanner, ils se traduisent par une image arrondie ou ovalaire hypodense ou isodense, avec rehaussement rapide après injection du produit de contraste. Il peut exister un renforcement périphérique en anneau réalisant l'aspect classique en cocarde. En imagerie par résonnance magnétique, les tuberculomes et l'œdème périlésionnel sont hypointenses au parenchyme cérébral en séquence T1. L'injection de gadolinium retrouve l'aspect de rehaussement annulaire classique en TDM. En T2, ils se traduisent par un signal hypointense avec plusieurs hypersignaux punctiformes à l'intérieur de la lésion et une zone pérphérique irrégulière d'hypersignal correspondant à l'œdème. L'IRM est l'examen de choix pour le diagnostic des tuberculomes cérébraux. Elle permet surtout par rapport à la TDM d'apprécier l'extension et les différentes composantes de la lésion (centre nécrotique, capsule et œdème péri-lésionnel).

**Figure 1:**
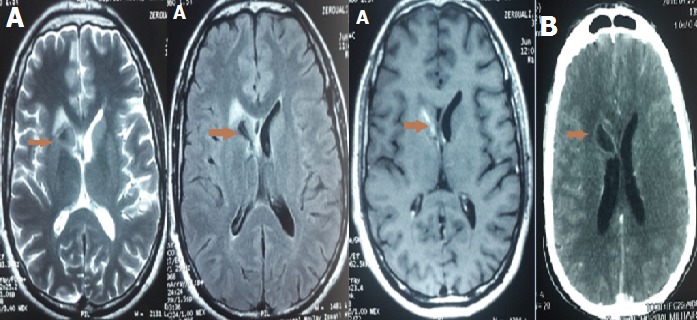
A): IRM cérébrale, en coupe axiale T2, FLAIR et T1 avec injection, montrant un tuberculome cérébrale du noyau caudé; B): TDM cérébrale avec injection, en coupe axiale, montrant un tuberculome du noyau caudé

